# *AFLPsim*: an R package to simulate and detect dominant markers under selection in hybridizing populations

**DOI:** 10.1186/1746-4811-10-40

**Published:** 2014-12-13

**Authors:** Francisco Balao, Juan Luis García-Castaño

**Affiliations:** Departamento de Biología Vegetal y Ecología, Universidad de Sevilla, Ap-1095, 41080 Sevilla, Spain; Department of Systematic and Evolutionary Botany, University of Vienna, Rennweg 14, Vienna, 1030 Austria

**Keywords:** Demographic simulation, Dominant markers, Genome scan, Hybridization, Outlier loci, R package

## Abstract

**Background:**

In spite of a large diversity of approaches to investigate loci under selection from a population genetic perspective, very few programs have been specifically designed to date to test selection in hybrids using dominant markers. In addition, simulators of dominant markers are very scarce and they do not usually take into account hybridization.

**Results:**

Here, we present a new, multifunctional, R package for dominant genetic markers, *AFLPsim*. This package can simulate dominant markers in hybridizing populations and implements genome scan methods for detecting outlier dominant loci in hybrids. In addition, it includes tools for further manipulating the results, plotting them and other tasks. We describe and tabulate the major functions implemented in *AFLPsim*. In addition, we provide some demonstration of its use and we perform a comparative study with other software. Finally, we conclude by briefly describing the input and output formats.

**Conclusions:**

The R package *AFLPsim* application provides several useful tools in the context of hybridization studies. It can simulate dominant markers in hybridizing populations and predict their demographic evolution. In addition, we implement a new genome scan method for detecting outlier dominant loci in hybrids, which shows a rather high sensitivity and is very conservative in comparison with Gagnaire *et al*.’s, Bayescan and *introgress*. The application is downloadable at http://cran.r-project.org/web/packages/AFLPsim/.

## Background

The study of natural hybridization has been the focus of much attention in Evolutionary Biology [1, 2]. Hybridization has recently been perceived as a catalyst not only for speciation but also for major evolutionary innovations [3]. Hybrid zones offer a “window on the evolutionary process” involving divergence at many loci by a balance between dispersal and selection [4]. Cline theory has provided a conceptual framework to understand the forces maintaining hybrid zones and to help infer the relevant evolutionary parameters describing the introgression of traits across hybrid zones [5]. However, in non-stable hybrid zones, selection in early generations has a central role in the establishment and fate of hybrids and progenitors [1, 4]. In these early generations, several hybrid categories can be easily distinguished (first generation hybrids –F_1_–, outcrosses between F_1_ individuals –F_2_–, or backcrosses to parental “A” –BxA– and backcrosses to parental “B” –BxB–, for instance). Hence, identifying markers under selection on these early-generated hybrids can provide tremendous knowledge about the stability of hybrid zones.

Although next generation sequencing (NGS) has transformed our ability to identify the genes underpinning selection/adaptation [6], a complementary approach with potentially neutral markers such as Amplified Fragment Length Polymorphisms (AFLPs) allows a cost-effective screening of the genomes of a large number of individuals [7]. AFLP has been very successfully used in the identification of hybrids [8, 9] and outlier loci presumably under selection [10–12]. However, in spite of a large diversity of approaches to investigate loci under selection from a population genetic perspective [13–15], no program has been specifically designed to date to test selection in hybrids using dominant markers. Nevertheless, genomic clines have been used to identify molecular markers with patterns of introgression inconsistent with neutrality (e.g. *introgress*[16, 17]). However, detecting outliers in a hybridizing framework using dominant markers can be a real challenge. Long periods of time are needed to identify loci that have experienced a history of weak selection, as cumulative effect is necessary to produce a detectable signal in the DNA polymorphism of the underlying loci [18]. Therefore, for hybrids in early generations, loci under weak selection would remain undetectable. In addition, dominance imposes difficulties to estimate allelic frequencies, especially when the frequency of the presence-allele is high [19]. Scoring errors and a low sample size can also affect the correct estimate of the allelic frequencies. Furthermore, previous methods used to detect dominant loci under selection have shown a substantial proportion of false positives among the detected outliers [20]. Hence, genome scan in hybrid zones should be capable to correctly estimate expected allelic frequencies under neutrality in hybrids overcoming these problems. Moreover, it should be sensitive enough to detect loci under moderate selection as well as keeping the false positive rate close to null.

Additionally, being able to simulate dominant markers in a hybrid zone is important to obtain a better knowledge about expected patterns of hybridization. When experimental treatments are not feasible, in-silico simulations have been widely used to test population genetic hypotheses [21]. Furthermore, genetic simulations have been also used to understand the statistical efficiency of several genetic methods and to compare different approaches [20]. Finally, simulations are of practical use when analysing data from a real system, as they can compare observed genetic distributions with the theoretically expected ones. In spite of its importance, simulators of dominant markers are very scarce [22] and they do not usually take into account hybridization, but see [23]. Again, dominance of markers is one of the major problems in the simulation process, as allele frequencies have to be estimated from phenotypic data.

Here, we describe *AFLPsim*, a software package designed to overcome these limitations by implementing a dominant marker simulator of hybridization and two genome scan algorithms (Gagnaire *et al*.’s [11] and a new method called bal&gar-ca) specifically designed to detect outlier markers in recent generated hybrids (F_1_, F_2_, BxA and BxB). The software is written in a statistical, open source, scripting R language [24], and released under the GPL license to guarantee the continuing availability of the source code.

## Implementation

A list of the major functions in the *AFLPsim* library is shown in Table 1. These functions cover methods in simulation, genome scan, and manipulation and visualization of results, which are detailed below. More details can be found in the software manual, which is available at https://github.com/fbalao/AFLPsim.

**Table 1:** **Functions of the** ***AFLPsim*** **package**

Function name	Description
*Simulations*	
demosimhybrid	conducts demographic analysis in hybrid populations.
hybridize	generates multilocus dominant hybrids individuals from parental profiles.
hybridsim	generates multilocus dominant parental and hybrid individuals.
*Genome scan*	
bayescan	calls Bayescan 2.1 program [12] from R to a set of populations.
gscan	conducts genome scan on F_1_ and backcross individuals [10].
hybridindex	estimates the hybrid index calling the *introgress* package.
*Plotting*	
plot.hybridsim	plots phenotypic frequencies of hybrids on a neutral hybridization model.
plot.demosimhybrid	plots results of ‘demosimhybrid’ function.
*Data manipulation*	
sim2adegenet	converts simulation to the *genind* format [25].
sim2arlequin	writes the input file for Arlequin[26] from the simulation results.
sim2bayescan	writes the input file for Bayescan[12] from the simulation results.
sim2popgene	writes the input file for Popgene[27] from the simulation results.
sim2introgress	converts simulation to the *introgress* format [16].
sim2newhybrids	writes the input file for Newhybrids[8] from the simulation results.
sim2structure	writes the input file for Structure[28] from the simulation results.

### Simulating hybridization and demographic evolution

Our software generates diploid hybrid genotypes, under the hypothesis of the Hardy-Weinberg equilibrium, Mendelian inheritance of markers and not linkage disequilibrium, by calculating observed allele frequencies in parental populations and the expected frequencies in the different hybrid classes (F_1_, F_2_ and backcrosses in both directions). Observed frequencies can be calculated from simulated parental populations with the ‘hybridsim’ function (following a beta distribution – [25]) or from two user-specified parental phenotypes (‘hybridize’ function), being the allele frequencies of dominant markers calculated using a square-root procedure [19]. For the F_1_ hybrids the expected phenotypic frequency of each band is
1

where *p*_A_ is the frequency of the presence-allele in the parental population A:
2

and *p*_B_ is the frequency of the presence-allele in the parental population B ().

For the other hybrid classes, we proceed in the same way. For example, for backcrosses with parental A, the expected frequency, based again on the parental allele frequencies, is
3

In addition, we implement phenotypic directional selection on the dominant allele, i.e. we modify the frequency of those individuals bearing a selected fragment regardless they are homozygous or heterozygous. Phenotypic selection on a specific marker is simulated with a conceptually simple variable (*s*), which ranges from -1 to +∞. This coefficient is 0 when there is no selection and it varies following negative and positive directional selection (negative and positive values, respectively). The expected frequency is calculated with the formula
4

where *w* is the ‘fitness’ , which relates to the selection coefficient (*s*) through the equation
5

Users can choose both the intensity of this coefficient (*s*) and the number of markers under selection.

The interplay of genetic and ecological processes often has important effects on the fate of the hybrid zones. Models can be powerful tools for investigating different control scenarios before undertaking expensive field trials. Plenty of mathematical models describing the dynamics and genetics of hybridization have been widely used. Briefly, these are categorised as ecological or genetic models. The advantages and disadvantages of these diverse approaches have been discussed in detail [26] and the utility of each model depends on the scenario and the supporting data. We implement a modified version of the genetic model of demographic evolution in hybrid zones (‘demosimhybrid’) developed by Epifanio & Philipp [27]. This heuristic model simulates the proportion of parentals, F_1_, F_x_ and backcrosses (with both parentals) individuals for each generation. The contribution of each taxon following admixture and hybridization depends on three independent variables: (1) the initial proportion of parental taxa; (2) the fitness gradient among parental and hybrid taxa; and, (3) the assortative mating between these taxa. Composition at any time (*t*, in generations) is calculated by multiplying its initial abundance by its relative fitness, and then, by the probability of mating, using the general equations 6 & 7.
6

where *S*_*G*_ is the proportion of a taxon *G* surviving to reproduction, *ϕ*_*t*_ is the frequency, before selection, of the taxon at the beginning of the generation *t*, and *ω*_*G*_ is its fitness. The expected contribution of a taxon to the subsequent generation is determined by the equation
7

where *M* is the assortative mating matrix.

Epifanio & Philipp’s model suffers from several pitfalls, e.g. migration is not taken into account and it is not spatially explicit [26, 28]. However, it has been successfully used to explain the extinction of progenitors in several hybrids zones [28, 29].

### Genome scan

*AFLPsim* performs two approaches for statistically seeking outlier loci (‘gscan’) in different hybrid classes (F_1_ and backcrosses). The first one, called ‘gagnaire’ [11] is based on a binomial test to assess any significant deviation between the observed and the expected frequencies for each marker. Briefly, Gagnaire *et al*.’s method estimates the frequency of the presence-allele based on one minus the square-root of the absence (null homozygote) frequency (Eq. 2). Therefore, using these parental frequencies (*f*_*A*_ and *f*_*B*_), expected band presence frequencies are then calculated for each hybrid category with the Eq. 1 (for F_1_ individuals) and the derived ones (e.g., Eq. 3). A binomial test is then performed to test for significant deviation between observed and expected frequencies of band presence at each locus in each hybrid category. For example, for F_1_ individuals:


where  is the observed frequency of band presence at one locus,  is the number of F_1_ hybrids and  is the expected frequency of band presence at that locus.

Using this method, some fragments could appear to be under selection when in fact they are not (i.e. false positives), because the observed parental frequencies (*f*_*A*_ and *f*_*B*_) can be biased by sampling error. For this reason, we implement a more conservative method (bal&gar-ca) calculating parental frequencies for each marker through √(1 - *α*) confidence intervals (in this case, *α* = 0.05) by the Clopper-Pearson ‘exact’ procedure, which is based on a beta distribution [30]. Every combination of one interval end from one parental and one interval end from the other parental lead to an expected value within the neutral expectation surface, and the four values delimit a 1 - *α* probability portion of it.


Applying Eq. 2 to these values we obtain the frequency intervals of the presence-allele in each parental (i.e. *p*_*LEA*_ , *p*_*UEA*_, *p*_*LEB*_, *p*_*UEB*_), and applying the Eq. 1, we finally obtain the four estimated values of the expected frequency of the presence-allele for F_1_ under neutrality (). For instance:


To test if a specific locus behaves as an outlier, the average real offspring value  is confronted against these four estimated values, considering the two following possibilities:

(i) If it is within the two most extreme values, we conclude the fragment is not under selection (i.e. ).
(ii) If not, we choose the closest of the four frequencies to be the expected value of the binomial test.

In both methods, the False Discovery Rate (FDR) correction is used to counteract for multiple comparisons and control for the expected proportion of the incorrectly rejected null hypotheses.

In addition, we also include a function that calls the Bayescan program [13] from R to perform a Bayesian estimation of selection; in this way, we facilitate the analysis and plotting of results of this efficient software. Moreover, the hybrid index (i.e. the genome-wide admixture) for the simulated hybrid individuals is calculated with the ‘hybridindex’ function. The maximum likelihood estimates (together with the 95% confidence intervals) of this hybrid index can be obtained with this function, which is a wrapper for the ‘est.h’ function of the package *introgress*[17].

### Data manipulation and visualisation

*AFLPsim* functions do not require external input files out of the R environment (Table 1). However, for the simulation of hybrids from user-specified parental data, these should be loaded to R as a *matrix* or a *data.frame*. Simulation results can be readily used by multivariate and phylogenetic methods of other R packages (e.g. *ade4*[31]; *adegenet*[32]). Our package is also able to export Arlequin formatted data [33] and Popgene[34] to estimate summary statistics (e.g. *F*-statistics, Shannon index or polymorphic loci) from the data set. Our package is also able to export data formatted for several popular population genetic computer programs such as Structure[35] and Newhybrids[9]. In addition, *AFLPsim* contains functions that produce graphics for visualising the expected frequencies under neutrality for loci under selection across the different hybrid classes. Finally, our package includes a function that plots the results of the demographic evolution model in a hybrid zone.

## Results and discussion

To demonstrate the capacities of *AFLPsim*, we assessed some comparisons on the behaviour of our genome scan method vs. that of other software. We have also created several illustrative examples, which can be easily reproduced. An example of the application of *AFLPsim* for investigating introgression patterns (bal&gar-ca method) and demographic dynamics (Epifanio & Philipp’s model) in a hybrid zone can be found in [28].

### Genome scan comparisons

We investigated the performance of our method (bal&gar-ca) under different scenarios using a simulation study and its efficiency was compared with that of Gagnaire *et al*.’s, Bayescan and *introgress*. Although Bayescan is not specifically designed for hybrids, we used it for comparison, as it is one of the most popular genome scan software. In this case, we used both parentals and F_1_ hybrids as three independent populations. *introgress* has been used to explore introgression between genomes through the genomic cline method. This method is able to identify those molecular markers with introgression patterns inconsistent with neutrality and, therefore, detecting possible loci under selection.

We performed three different simulation experiments to compare the outlier detection efficiency of different genome scan methods, different selection coefficients, different sample sizes and different percentage of selected loci in the genome. We carried out all the simulations on the University of Oslo Bioportal (http://www.bioportal.uio.no) using the ‘hybridsim’ function.

*1) Comparison of different genome scan methods*

We compared the results obtained with four methods (bal&gar-ca, Gagnaire *et al*.’s, *introgress* and Bayescan), with three selection coefficients (*s* = 2.162, log (*s* + 1) = 0.5, weak selection; *s* = 99, log (*s* + 1) = 2, strong selection; *s* = 999, log (*s* + 1) = 3, very strong selection). 100 F_1_ hybrid individuals were simulated with ‘hybridsim’ from two parental populations (A and B) of 100 individuals each one. We simulated 1000 independent loci (i.e. based on linkage disequilibrium), where 100 were introgressed under directional selection in the F_1_. Every scenario (i.e. every selection coefficient) was replicated 100 times. Genome scans were performed on simulations with our method (bal&gar-ca), that by Gagnaire *et al*., *introgress* and Bayescan 2.1, assessing the ability of each one for detecting selection of different intensities as well as the proportion of failures as false positives.

Results showed, on the one hand, that our method was very conservative and that its sensitivity (i.e., the rate of true positives) was lower than that by Gagnaire *et al*. [11] and *introgress*; on the other hand, Bayescan almost invariably failed to detect outliers for the simulated datasets (Table 2). However, our method had a null false positive rate unlike Gagnaire *et al*.’s and *introgress* (~15% and ~13% false outlier detection, respectively).

**Table 2:** **Summary of sensitivity (true positive rate ± SD) and type I error rate (false positive rate ± SD) for outlier methods tested with 100 simulated data for three regimes of divergent selection**

Method	Weak selection (***s*** = 2.162)		Strong selection (***s*** = 99)		Very strong selection (***s*** = 999)	
	Sensitivity	Type I error	Sensitivity	Type I error	Sensitivity	Type I error
bal&gar-ca	0.124 ± 0.039	0.000 ± 0.000	0.479 ± 0.050	0.000 ± 0.000	0.487 ± 0.049	0.000 ± 0.000
Gagnaire *et al*.’s	0.572 ± 0.052	0.147 ± 0.012	0.621 ± 0.048	0. 149 ± 0.012	0.619 ± 0.049	0.147 ± 0.013
*introgress*	0.454 ± 0.034	0.127 ± 0.015	0.717 ± 0.061	0.126 ± 0.010	0.723 ± 0.052	0.128 ± 0.014
Bayescan	0.025 ± 0.021	0.029 ± 0.001	0.035 ± 0.019	0.012 ± 0.004	0.080 ± 0.019	0.037 ± 0.005

*2) Impact of different scenarios on outlier detection*

In the second approach we compared specifically our method with Gagnaire *et al*.’s and we tested the effect of the sampling bias, selection coefficient and percentage of loci under selection on the detection of outliers. A range of possible scenarios were run regarding: (i) different parental sampling sizes (3, 5, 10, 30, 50, 100, 300, 500 and 1000 individuals, from the original populations of 1000 individuals each) for a *s* = 99 [log (*s* + 1) = 2]; (ii) different values of the selection coefficient (-0.999, -0.990, -0.900, -0.684, 0.000, 2.162, 9.000, 99.000 and 999.000; equivalent to log (*s* + 1) values of -3.00, -2.00, -1.00, -0.50, -0.25, -0.10, -0.05, 0.00, 0.05, 0.10, 0.25, 0.50, 1.00, 2.00 and 3.00, respectively) with a parental sampling of 100 individuals each (from the original populations of 1000 individuals each); (iii) different proportions of selected loci from the 1000 simulated ones (0.0%, 0.5%, 1.0%, 5.0% and 10.0%). Every scenario was replicated 100 times.

**Figure 1:**
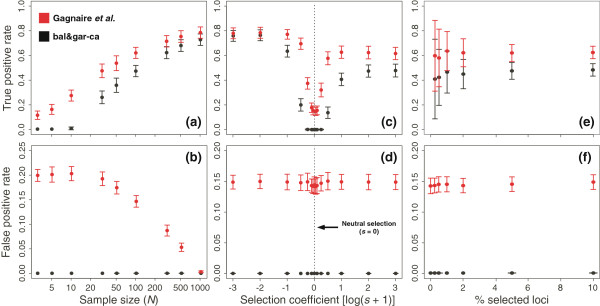
**Effect of the parental sample size (** ***N*** **) on (a) the detection of true outlier loci and (b) rate of false positives, for the two methods.** Influence of the selection coefficient (*s*) on **(c)** the detection of true outlier loci and **(d)** rate of false positives. Effect of the percentage of selected loci on **(e)** the detection of true outlier loci and **(f)** rate of false positives. Simulations (*N* =100) were performed for 900 neutrally introgressed loci and 100 loci under selection for Gagnaire *et al.*’s (in red) and bal&gar-ca (in black) methods. Bars for ± standard deviation values.

Results (Figure 1) showed, on the one hand, that the bal&gar-ca method was very conservative and that its sensitivity (i.e., the rate of true positives) was lower than Gagnaire *et al*.’s. However, the bal&gar-ca method had a null false positive rate unlike Gagnaire *et al*.’s (~15% false outlier detection). Regarding the sampling bias (affecting the estimates of parental frequency), although the sensitivity (Figure 1a) of the bal&gar-ca method is specially lower than Gagnaire *et al*.’s at low sample sizes (i.e. with a high deviation in the parental frequency estimates), in these cases Gagnaire *et al*.’s suffers from a much higher false positive rate (Figure 1b). Both methods behave in a similar way in relation to varying selection coefficients and percentage of selected loci (Figure 1c–f). In our simulation, the sensitivity of both methods decreases at low values of the selection coefficient. Although detection of loci under selection becomes null in the bal&gar-ca method, its rate of false positives is null as well. Otherwise, the type I error rate of Gagnaire *et al.*’s method remains about 15% regardless of the selection coefficient values. Lastly, the mean rates of true and positive values obtained by both methods are not affected by the percentage of selected loci (Figure 1e–f). However, when the percentage of selected loci increases, the standard deviation of the sensitivity values decreases.

*3) Effect of the frequency estimation method*

The original formulation of Gagnaire *et al*.’s and bal&gar-ca methods use the square-root method to estimate the null-allele frequency. As the allele frequency estimation is difficult in dominant markers, we investigated the effect on the outlier detection of using a Bayesian estimator of the null-allele frequency, , with non-uniform priors [36]. For the bal&gar-ca method, we also modified the calculation of the frequencies of the parental allele confidence intervals, by using a quantile-based 95% probability interval based on the Bayesian estimator of the squared standard error, [36]. To compare this Bayesian method and the previous approach for a particular common scenario, 900 independent loci plus 100 selected loci (*s* = 99) were simulated in 100 F_1_ hybrid individuals from two parental populations (A and B) with 100 individuals each. One hundred simulations were carried out to compare the results of both methods (Gagnaire *et al*.’s and bal&gar-ca) with both different allelic frequencies estimation (square-root and Bayesian).

**Figure 2:**
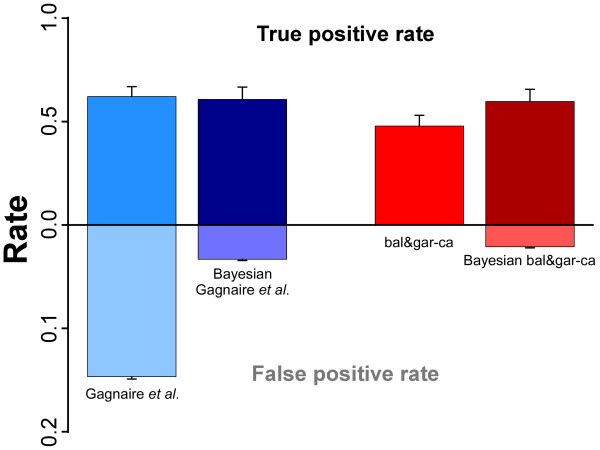
**Effect of the allele frequency estimation method (square-root and Bayesian estimations –non-labelled and labelled columns, respectively) on the detection of true outlier loci and rate of false positives for Gagnaire** ***et al.*** **’s and bal&gar-ca methods.** Bars for the standard deviation.

Figure 2 shows the results for different allelic frequency estimates in the two genome scan methods. Bayesian estimation has strong effect in both methods. For Gagnaire *et al*.’s method, using Bayesian estimations slightly decreased its sensitivity, but it highly decreased its rate of false positives (from 15% to 4%). For the bal&gar-ca method, using Bayesian estimations improved its sensitivity, but its rate of false positives also increased (reaching almost 3% –which, nevertheless, was not very high).

In stable hybrid zones with several generations of hybrids, *introgress* seems to be a compromise solution. However, in transitory recent hybrid zones, bal&gar-ca and Gagnaire *et al*.’s methods are better options, depending on the size of the hybrid zone, our sampling strategy and the scope of the genome scan. Gagnaire *et al*.’s is useful when we have a good population sampling. However, when the sample sizes are modest and we want to avoid any possible false positive, bal&gar-ca is the advised method. Although Bayesian estimation of frequency allele has been proved to be useful in many cases, it is not advised for the bal&gar-ca method as it increases its type I error rate. Finally, none of these methods is advisable when the selection coefficient shows low values, which is not strange as, in these cases, long periods of time would be needed to accumulate detectable signal in the DNA [18].

### Simulating hybridization with selection and genome scan for F_1_ individuals

In this example, we carry out the simulation of two parental populations of 100 individuals and 100 F_1_ hybrids for a total of 300 markers using the ‘hybridsim’ function. Positive selection was simulated with *s* = 10 for 15 out of 300 markers.

Firstly, we need to load *AFLPsim* and set the random seed number (arbitrarily to 123) for reproducibility.

> require(AFLPsim)

> set.seed(123)

> f1hybrid<-hybridsim(Nmarker=300, Na=100, Nb=100, Nf1=100, + type = 'selection', hybrid = 'F1', S=10, Nsel=15)

This is an object ‘hybridsim’ that contains the presence-absence matrices for the parentals (PA and PB) and for the hybrid classes (in this case, F_1_). In addition, this object contains the loci under selection (f1hybrid$SelMarkers) and the selection coefficient used in the simulation (f1hybrid$S).

> f1hybrid$F1

$F1

M1 M2 M3 …

F1_1 0 1 0 …

F1_2 1 1 0 …

.

.

.

> f1hybrid$SelMarkers

[1] 13 26 46 55 69 78 149 158 161 177 183 229 230 289 290

> f1hybrid$S

[1] 10

**Figure 3:**
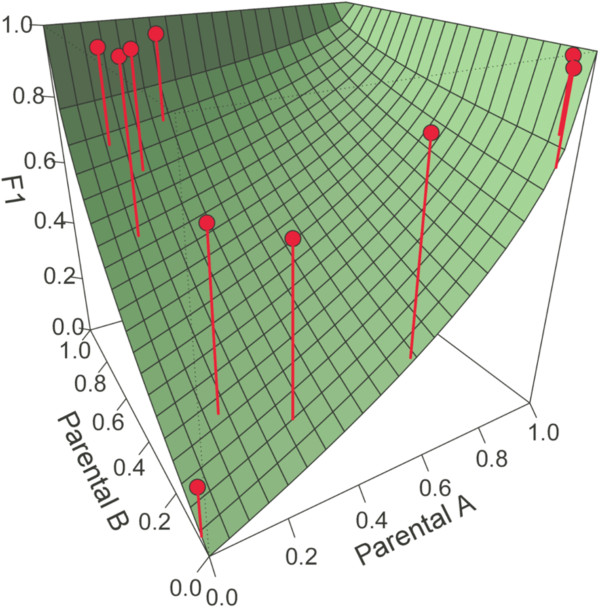
**Three-dimensional scatter plots showing significant outlier loci detected by the ‘gscan’ function for the simulated F**_**1**_ **hybrids.** The green-coloured surface shows the theoretical probability of observing a dominant marker as a function of the band presence frequency in each parental species. The difference between the observed and the theoretical band frequency is represented with a vertical line joining both values.

Then we perform a genome scan with the bal&gar-ca method setting the type parameter to the correct hybrid class (i.e. F_1_ hybrids). The results of the genome scan analysis are saved in a data object (outlier), which includes the *P*-values of the binomial test after FDR (outlier$fdrf1), and the loci identity of those markers with *P* < 0.05 (outlier$Outliers). Moreover, this object is used as the input for the ‘plot.hybridsim’ function to generate a plot of the outlier markers and the expected frequencies under neutrality (Figure 3).

> outlier<-gscan (f1hybrid, type = 'F1', method='bal&gar-ca')

> str (outlier)

List of 2

$ Pvalues :'data.frame': 300 obs. of 1 variable:

..$ fdrf1: num [1:300] 1 1 1 1 1 1 1 1 1 1 …

$ Outliers: num [1:10] 26 46 55 69 149 161 177 229 230 289

> plot.hybridsim(f1hybrid,hybrid = 'F1', + markers=outlier$Outliers)

In this example, we detected 10 out of 15 loci under selection (67% sensitivity) and we did not obtain any false positive.

### Simulating demographical evolution under hybridization

Finally, we simulated hybridization on one area and evaluated its demographical consequences. For the initial frequencies we created a vector with the frequencies of Parental A, Parental B, F_1_, Backcross to Parental A, Backcross to Parental B and F_x_. In our case, we fix Parental A and Parental B initial frequencies to 0.5.

> freqinit<-c (0.5,0.5,0,0,0,0)

Then, we create a matrix of assortative mating using the matrix function, and allow crosses between all taxa with the same probability.

> matingmat<-matrix (1,ncol=6,nrow=6)

**Figure 4:**
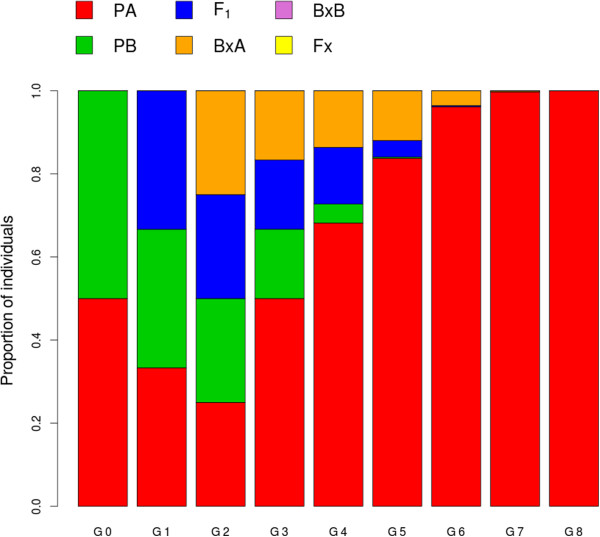
**Simulated demographic evolution of a hybrid zone under similar initial proportions of the parentals, using a modified version of Epifanio & Philipp's model.** Each bar represents the relative proportion of each parental and hybrid category (see legend) in the area over 8 generations (G0-G8), until Parental A (PA) dominates the area.

In this example, parentals have similar fitness but F_1_ individuals’ is lower than parentals’. Here, we want to force asymmetrical introgression and breakdown occurs after F_1_ hybrids, with posterior hybrid generations (F_x_) and backcrosses to Parental B being sterile, whereas backcrosses to Parental A (BxA) would have a similar fitness to F_1_ individuals. Hence, fitness would be modified as following:

> fitness<-c (1,1,0.5,0.5,0,0)We obtain a matrix with the frequency of each taxon in eight generations. Parental A dominates the hybrid zone after eight generations, and displaces the other parental and the hybrids. We used the ‘plot.demosim’ function to visualise this demographic evolution (Figure 4):

> set.seed(123)

> results<-demosimhybrid(freqinit, matingmat, fitness)

> results

PA PB F1 BPA BPB Fx

G0 0.500 0.500 0.000 0.000 0 0

G1 0.333 0.333 0.333 0.000 0 0

G2 0.250 0.250 0.250 0.250 0 0

G3 0.500 0.167 0.167 0.167 0 0

G4 0.682 0.045 0.136 0.136 0 0

G5 0.838 0.003 0.040 0.120 0 0

G6 0.962 0.000 0.002 0.036 0 0

G7 0.998 0.000 0.000 0.002 0 0

G8 1.000 0.000 0.000 0.000 0 0

attr(,"class")

[1] "demosim.hybrid"

> plot.demosimhybrid (results)

## Conclusions

This simulation study showed the interest of performing comparative studies in hybridization analytical software. Here we fill an important gap in this kind of software, as the R package *AFLPsim* application provides several useful tools in the context of hybridization studies. This is true specifically in relation to dominant markers with low sample sizes, in order to obtain markers under selection with a low rate of false positives and with a rather high sensitivity. None of the methods used is advisable when the selection coefficient shows low values. Moreover, *AFLPsim* provides a demographic method to study evolution in this context. Finally, we hold an on-going project to implement the bal&gar-ca method with biallelic codominant markers as well as single nucleotide polymorphisms (SNPs).

### Availability and requirements

**Project name**: *AFLPsim*

**Project home page**: http://cran.r-project.org/web/packages/AFLPsim/

**Operating system(s)**: Windows, Mac OS, Linux

**Programming language**: R

**Other requirements**: R version 2.15 or higher

**License**: GPL-2 | GPL-3

**Any restrictions to use by non-academics**: None
